# Proteomic profiling of proteins in the dorsal horn of the spinal cord in dairy cows with chronic lameness

**DOI:** 10.1371/journal.pone.0228134

**Published:** 2020-01-28

**Authors:** Daniel Herzberg, Pablo Strobel, Heine Müller, Constanza Meneses, Marianne Werner, Hedie Bustamante

**Affiliations:** 1 Veterinary Clinical Sciences Department, Faculty of Veterinary Science, Universidad Austral de Chile, Valdivia, Chile; 2 Animal Science Department, Faculty of Veterinary Science, Universidad Austral de Chile, Valdivia, Chile; 3 Comparative Biomedical Science Graduate Program, College of Veterinary Medicine, North Caroline State University, Raleigh, North Carolina, United States of America; Pacific Northwest National Laboratory, UNITED STATES

## Abstract

Chronic lameness affects bovine welfare and has a negative economic impact in dairy industry. Moreover, due to the translational gap between traditional pain models and new drugs development for treating chronic pain states, naturally occurring painful diseases could be a potential translational tool for chronic pain research. We therefore employed liquid chromatography tandem mass spectrometry (LC-MS/MS) to stablish the proteomic profile of the spinal cord samples from lumbar segments (L2-L4) of chronic lame dairy cows. Data were validated and quantified through software tool (Scaffold® v 4.0) using output data from two search engines (SEQUEST® and X-Tandem®). Search Tool for the Retrieval of Interacting Genes/Proteins (STRING) analysis was performed to detect proteins interactions. LC-MS/MS identified a total amount of 177 proteins; of which 129 proteins were able to be quantified. Lame cows showed a strong upregulation of interacting proteins with chaperone and stress functions such as Hsp70 (*p* < 0.006), Hsc70 (*p* < 0.0079), Hsp90 (*p* < 0.015), STIP (*p* > 0.0018) and Grp78 (*p <*0.0068), and interacting proteins associated to glycolytic pathway such as; γ-enolase (*p* < 0.0095), α-enolase (*p* < 0.013) and hexokinase-1 (*p* < 0.028). It was not possible to establish a clear network of interaction in several upregulated proteins in lame cows. Non-interacting proteins were mainly associated to redox process and cytoskeletal organization. The most relevant down regulated protein in lame cows was myelin basic protein (MBP) (*p* < 0.02). Chronic inflammatory lameness in cows is associated to increased expression of stress proteins with chaperone, metabolism, redox and structural functions. A state of endoplasmic reticulum stress and unfolded protein response (UPR) might explain the changes in protein expression in lame cows; however, further studies need to be performed in order to confirm these findings.

## Introduction

Chronic pain represents a dysfunction of the nervous system [1, 2], and similar to humans, this type of pain impact negatively the quality of life of affected animals [3]. Naturally occurring painful diseases in animals may represent an alternative approach to investigate nociceptive mechanisms involved in chronic pain [4, 5]. Painful lameness in dairy cows is common, and one of the most important causes of economic losses for the dairy industry [6] due to increasing culling [7]https://paperpile.com/c/QlNkzH/hxba, reduced milk production and reproductive performance [8]. https://paperpile.com/c/QlNkzH/Hj7c+4D5eLameness is a multifactorial condition and its prevalence has been associated with housing and nutritional management [9]https://paperpile.com/c/QlNkzH/Cn8P+388y+UI6B+9Lth, environmental factors [10]https://paperpile.com/c/QlNkzH/6tmv, metabolic status [11], inadequate claw trimming [9], gestation and stage of lactation [11]. The characteristic features of chronic lameness associated-pain in dairy cows make this condition a potential new translational model for the study of chronic pain.

Within the damaged tissue, inflammatory mediators released from immune cells can reduce the threshold of somatic and visceral primary afferent nociceptors, leading to peripheral sensitization [12]https://paperpile.com/c/QlNkzH/2xFo+o6FS. Prostanoids [13], kinins [14], growth factors [15], chemokines [16], cytokines [17], protons and ATP [18] can promote peripheral sensitization, activating multiple intracellular transduction signals that lead to an increase in membrane receptor expression (i.e., TRPV1, TRPVA1, Nav1.7, Nav1.9, among others). Moreover, persistent peripheral sensitization can potentially induce a state of central sensitization [12]. Central sensitization is the facilitation of synaptic transmission in central nervous system (CNS), which often turns into maladaptive and prolonged molecular changes in the nociceptive pathway [1]. This increased sensitivity to noxious and harmful stimuli will create pain behaviors known as hyperalgesia and allodynia, respectively [19]. It has been previously demonstrated that reduced nociceptive umbral thresholds can be detected in cattle with mild to severe lameness associated to chronic inflammatory lesions of the hoof [20].

Two temporal phases can be distinguished during central sensitization: an early phase dependent on kinase activation that results from rapid changes in the glutamate receptor and ion channels function [21, 22], and a later and long-lasting phase in which transcriptional and translational events drive the synthesis of proteins responsible for persistent pain [1, 23].

Proteins expression can be determined through proteomic analysis [24]. Proteomic techniques have been used in order to investigate the dynamics of protein expression under pathological pain states, with an increased potential for identification of pain biomarkers [25]. Most of the proteomic studies focusing on pain have been performed either in the spinal cord or in the dorsal root ganglion (DRG) of rodents using experimental pain models [26]. Recently, increasing concern about the translational impact of basic science research in the development of new drugs has been discussed [25]. According to Mao (2012) [27], the time frame of pain in experimental models may not adequately reproduce the impact of prolonged nociception of clinical pain. Moreover, few proteomic studies have focused on naturally occurring pathological pain. Recently, the cerebrospinal fluid, serum and plasma proteome of human patients with neuropathic pain, rheumatoid arthritis and widespread back pain has been described [28, 29, 30].

The aim of this study was to describe the proteomic profile in the dorsal horn spinal cord of cows with chronic inflammatory lameness.

## Materials and methods

### Bioethics statement

The experimental protocol was approved by the Ethics Committee of Animal Research of the Universidad Austral de Chile (resolution number 323/2018).

### Animals

Twelve dairy cows were selected from a commercial dairy farm (Agricola Los Ríos, single livestock role 10.5.07.0760). All selected animals were Kiwi cross and 2 years of age or older, originating in different herds within the same farm with similar breeding, feeding and other routine practices. Spinal cord samples from 5 lame dairy cows (Lame group; n = 5) with a hind limb lameness history of at least 5 months were obtained after euthanasia via intravenous general anesthesia and intrathecal lidocaine injection at the atlanto-occipital foramen as previously reported. Similarly, spinal cord samples from 7 non-lame animals (Control Group; n = 7) without apparent lameness and without history of previous lameness episodes were selected from a commercial slaughterhouse (Frigorifico Balmaceda SPA, single livestock role 10.512.0882) after euthanasia by mechanical stunning and exsanguination according to national regulations. Researchers were not involved in the decision for euthanasia or slaughter.

### Lameness assessment

Given that bovine lameness is a multifactorial condition, cows were selected considering the most prevalent causes of lameness in Southern Chile, including white line disease, sole hemorrhage, sole ulcer and digital dermatitis [31]. Lameness was confirmed and classified according to the mobility score previously described by Reader et al. (2011) [32]. Briefly, cows were classified into two groups (Lame or Control), and lameness was scored as follow: MS 0 not lame; MS 1 imperfect mobility/uneven; MS 2 impaired mobility/mildly lame; and MS 3 severely impaired mobility/very lame. Lame group consisted only of cows with a MS of 3. Exclusion criteria for both groups included the presence of visible acute wounds, visible neurological gait alteration (central or peripheral ataxia) and acute or chronic mastitis.

### Spinal cord sampling and protein extraction

Lumbar spinal cord sections (L2-L4) were aseptically obtained post-mortem after removal of the dorsal aspect of lumbar vertebrae. A 20 cm segment was obtained from each animal. Dura mater and arachnoids meninges were gentle dissected and after carefully washing the tissue with cold phosphate buffered saline (PBS) samples were snap frozen in liquid nitrogen and transported to laboratory for further processing. Several segments of approximately 300 mg of the ipsilateral dorsal horn were stored in a mixture of 1 mL of PBS and 10 uL of protease inhibitor. For protein extraction, samples were sonicated three times for 30 seconds each and then centrifuged at 20,000 x g for 20 min at 4°C. The supernatant was collected and stored at -80°C until further analysis. Total protein quantification was performed using the BCA Assay (Pierce^TM^ Thermo Scientific, Rochford USA) according to the manufacturer's instructions. Protein extraction was evaluated using a 5–12% sodium dodecyl sulfate polyacrylamide gel electrophoresis (SDS-PAGE) stained with Coomassie blue.

### Sample preparation for proteomics analysis

100 μg of protein were lyophilized for 120 minutes and then re-solubilized in 6M guanidine hydrochloride and 25 mM NH_4_HCO_3_ at pH 7.5 during 60 minutes. Subsequently, proteins were reduced at room temperature using 2 mM dithiothreitol and alkylated with 10 mM iodoacetamide at room temperature for 60 minutes. These reactions were then diluted seven times with 25 mM NH_4_HCO_3_ at pH 7.5. Modified trypsin 1:50 (Promega, Madison, WI, USA) was added, and the reaction was incubated at 37°C for 16 hours. Trypsin digestion was stopped adding 5% formic acid, pH 3.0.

### Protein identification by tandem mass spectrometry (LC-MS/MS)

Protein samples were concentrated using a CentriVap concentrator (Labconco, Kansas City, MO, USA) to a final volume of 20 μL and loaded into a 350 μm internal diameter (ID) fused silica peptide trap column with 3 cm of C18 reverse-phase desalting resin. Immediately after washing with 0.1% formic acid for 30 min at 0.5 μL/min, the efflux of the peptide trap column was directed to a 10 cm resolving reversed-phase column (100 Å, 5 μm Magic C18 particles, Michrom Bioresources), which was mounted on the electrospray stage of a VELOSPRO mass spectrometer (LTQ, Thermo Finnigan LLC) by a 0–90% acetonitrile gradient for 120 min at a flow rate of 600 nL/min. An electrospray voltage of 2.2 kV with the ion transfer temperature set to 270ºC was used. The mass spectrometer was controlled by the Xcalibur software, which continuously performed mass-scan analysis of the six most intense ions during MS/MS scans of the ion traps. For this, one repeat scan of the same ion was dynamically excluded, using a 30 sec repeat duration and 90 s exclusion duration. Normalized collision energy for the MS/MS was set to 35%.

#### Database search algorithm and protein identification criteria

SEQUEST (Thermo Fisher Scientific, San Jose, CA, USA) and X!Tandem software (www.thegpm.org) were used to analyse all tandem mass spectra MS/MS samples. SEQUEST was set up to search Bostauros_NCBI_250722016.fasta (60.090 entries) with the inclusion of trypsin. Similarly, X!Tandem was set up to search a reverse concatenated subset of the uniprot-bos+taurus database (200 entries) also assuming trypsin digestion. Both databases were searched with a fragment ion mass tolerance of 0.80 Da and a parent ion tolerance of 2.5 Da. Carbamidomethyl of cysteine was specified in SEQUEST and X!Tandem as a fixed modification. Deamidated of asparagine and glutamine and oxidation of methionine were specified in SEQUEST as variable modification. Glu→pyro-Glu of the n-terminus, ammonia-loss of the n-terminus, gln→pyro-Glu of the n-terminus, asparagine and glutamine deamidation, methionine oxidation were specified in X!Tandem as variable modifications.

In order to validate MS/MS peptide and protein identification, data were loaded into Scaffold v.4.8.6 (Proteome Software Inc., Portland, OR). Peptide and Protein probabilities were assigned using the peptide/protein prophet algorithm [33]. Peptide and protein criteria of identification included a 95% confidence in the peptide/protein prophet algorithm, with a minimum of 4 identified peptideshttps://paperpile.com/c/QlNkzH/LDZX [34]https://paperpile.com/c/QlNkzH/oLuB. Proteins that contained similar peptides and could not be differentiated based on MS/MS analysis were grouped to satisfy the principles of parsimony. Proteins sharing significant peptide evidence were grouped into clusters. Proteins were there annotated with gene ontology (GO) terms from goa uniprot_all.gaf (downloaded Jan 12, 2017) [35]. Unique peptide counts are included as supplemental information (Supp 1).

Additionally, a search tool for the retrieval of interacting genes/proteins (STRING) neighborhood analysis was performed on all identified proteins by searching the STRING database to detect possible interactions (https://string-db.org). Protein names were obtained by matching the gen-info identifier number (GI) of the proteins to the UniProtKB database (https://www.uniprot.org). STRING was set to identify the evidence of the type of interaction, selecting database and experiments as active interaction sources and a minimum required interaction score of 0.900 [36].

### Western blot analysis

Western blot analysis was performed in order to validate some proteins of interest detected in proteomics. Protein extraction was performed using the same method described previously for LC-MS/MS. Proteins were quantified by BSA Assay (Pierce^TM^ Thermo Scientific Rockford USA) and twenty microgram of each sample was separated by SDS-PAGE and transferred to a 0.45 μm nitrocellulose membrane (Amersham^™^ Potan^™^ GE Healthcare Germany). Membranes were blocking during 1 hour with TBS (10 mM Tris-HCl pH 7,6) and 5% BSA at room temperature. Membranes were incubated for 16 h at 4°C with the following antibodies: mouse monoclonal anti gamma-enolase-enolase (1:500 w/v, Santa Cruz Biotechnology), mouse monoclonal anti-Hsp70 (1:500 w/v, Santa Cruz Biotechnology) and mouse monoclonal anti-β-actin (1:500 w/v, Santa Cruz Biotechnology) as loading control. Following primary antibody incubation, membranes were washed three times (10 minutes each) with TBS and 0.1% Tween-20, and incubated during 60 minutes at room temperature with anti-mouse IgG HRP-secondary antibody (1:2000 v/w, Santa Cruz Biotechnology). Following secondary antibody reaction membranes were washed three times for 10 minutes each. The intensity of the bans were visualized with enhanced chemiluminescence (PierceⓇ ECL Thermo Scientific Rockford USA) and captured by Odyssey Imaging System (LI-COR Bioscience, Lincoln, NE). For quantification of blot intensities Image Studio Lite ver 5.2.5 software (LI-COR Bioscience, Lincoln, NE) was used.

### Statistical analysis

Label free total spectra counting and total abundance of proteins for lame and control cows were tested for normality using the Kolmogorov-Smirnov test and then compared between groups using the *t*-test. Band intensities, expressed as mean ±SEM, were tested for normality using the Kolmogorov-Smirnov test and then compared between groups using the *t*-test. Differences were considered significant when p ≤ 0.05. All statistical tests were performed on Graphpad Prism 7.0.

## Results

Tandem mass spectrometry analysis was able to identify a total amount of 177 proteins in the dorsal horn of the spinal cord. Proteins with significant peptide evidence were grouped into 47 clusters. From the totality of identified proteins, Scaffold software was able to quantify 129 of them. Quantitative analysis showed that 10 proteins were significantly downregulated, and 27 proteins were significantly upregulated in cows with chronic lameness compared to controls (Tables 1 and 2). Moreover, 26 proteins were only detected in the dorsal horn of the spinal cord of lame cows (Table 2).

**Table 1:** Downregulated proteins detected in the spinal cord of lame cows.

Proteins	Accesion (GI)	MW (kDa)	Gene Name	*p-*value	Fold Change
Albumin	ALBU_BOVIN	69	**ALB**	0,0033	0,3
Dihydropyrimidinase-related protein 2	528959240	65	**DPYSL2**	0,0013	0,6
Glyceraldehyde 3-phosphate Dehydrogenase	694270100	38	**GAPD**	0,036	0,7
Hemoglobin beta	294459577	16	**HBB**	0,00016	0,2
Hemoglobin beta Bali	223864			0,00028	0,3
Hemoglobin chain C	97724899			0,001	0,5
Hemoglobin fetal subunit beta	62460494	15	**HBG**	0,00013	0,3
Hemoglobin subunit alpha	359061887	15	**HBA**	0,0022	0,3
Myelin basic protein	741972060	33	**MBP**	0,02	0,7
Ubiquitin carboxyl-terminal hydrolase	528952847	25	**UCHL1**	0,0018	0,4

**Table 2:** Upregulated proteins detected in the spinal cord of lame cows.

Proteins	Accesion (GI)	MW kDa	Gene Name	*p* value	Fold Change*
4-aminobutyrate aminotransferase, mitochondria	125991950	56	**ABAT**	0,0012	5,9
4-trimethylaminobutyraldehyde dehydrogenase	114051782	54	**ALDH9A1**	0,017	6,1
78 kDa glucose-regulated protein	115495027	78	**GRP78**	0,0068	INF
Acyl-CoA-binding protein	ACBP_BOVIN	10	**DBI**	0,048	3,1
Cdc42	7245833	25	**CDC42**	0,0001	INF
Acetyl-CoA acetyltransferase, mitochondrial	114050959	45	**ACAT1**	0,038	INF
Aconitate hydratase	27806769	85	**ACO2**	0,045	4,3
AHNAK2	983004191	186	**AHNAK2**	0,0025	8,3
Aldehyde dehydrogenase, mitochondrial	115496214	57	**ALDH2**	0,012	11
Aldose reductase	113594	36	**AKR1B1**	0,029	INF
Alpha-aminoadipic semialdehyde dehydrogenase	296485604	59	**ALDH7A1**	0,014	INF
Arylsulfatase B precursor	155372077	59	**ARSB**	0,00026	INF
Aspartate aminotransferase, cytoplasmic	29135295	?	**GOT2**	0,00063	19
Aspartate aminotransferase, mitochondrial	27807377	48	**GOT1**	0,0049	4,9
ATP-citrate synthase	82697335	17	**ACLY**	0,032	17
Dihydrolipoyl dehydrogenase, mitochondria	329663954	54	**DLD**	0,0011	INF
Dihydropyrimidinase-related protein 1	741930532	74	**CRMP1**	0,0053	INF
Dihydropyrimidinase-related protein 3	155371867	74	**DPYSL3**	0,00082	9,9
Enolase 1	296479148	47	**ENO1**	0,013	1,9
Enolase 2	528950986	47	**ENO2**	0,0095	1,8
Enolase 3	77736349	47	**ENO3**	0,011	2
Fascin	78045491	58	**FSCN1**	0,00045	21
Galectin-1	999589	14	**LGALS1**	0,024	1,9
Glutathione S-transferase P	29135329	24	**GSTP1**	0,017	6,7
Heat shock 70 kDa protein 1	529003643	72	**HSPA1L**	0,006	INF
Heat shock 70 kDa protein 4	166795319	70	**HSPA4**	0,006	INF
Heat shock 70 kDa protein 6	297472417	70	**HSPA6**	0,0029	INF
Heat shock cognate 71 kDa protein	296480084	70	**HSPA8**	0,0079	24
Heat shock protein HSP 90-alpha	60592792	90	**HSP90AA1**	0,015	INF
Heat shock-related 70 kDa protein 2	296482938	70	**HSPA2**	0,0063	INF
Hexokinase-1	60592784	102	**HK1**	0,028	INF
Hyaluronan and proteoglycan link protein 2	528942294	38	**HAPLN2**	0,00047	14
L-isoaspartate(D-aspartate) O-methyltransferase	296483921	25	**PCMT1**	0,036	10
Microtubule-associated protein 1A	741967576	336	**MAP1A**	0,0019	INF
Microtubule-associated protein 1B	329663571	330	**MAP1B**	0,0002	INF
N(G),N(G)-dimethylarginine dimethylaminohydrolase	156121049	31	**DDAH1**	0,0014	4,9
Peroxiredoxin-1	296488840	22	**PRDX1**	0,0039	5,3
Peroxiredoxin-4	27806085	31	**PRDX4**	0,0001	INF
Phosphoglucomutase-1	116004023	62	**PGM1**	0,0002	6,4
Pyridoxal phosphate phosphatase	78045487	32	**PDXP**	0,016	INF
Rab GDP dissociation inhibitor alpha	27806617	51	**GDI1**	0,031	INF
Rab GDP dissociation inhibitor beta	76253900	50	**GDI2**	0,022	22
retinal dehydrogenase 1	27806321	55	**ALDH1A1**	0,014	
Stress-70 protein, mitochondrial	77735995	74	**HSPA9**	0,008	INF
Stress-induced-phosphoprotein 1	296471478	63	**STIP1**	0,0018	INF
Superoxide dismutase [Cu-Zn]	SODC_BOVIN	16	**SOD1**	0,021	4,3
Tenascin	528959916	260	**TNC**	0,0001	INF
Transferrin	602117	24	**TF**	0,034	24
Transketolase	148744821	68	**TKT**	0,033	8,6
Versican	296485061	370	**VCAN**	0,0046	3,6
Zeta-crystallin	4097831	35	**CRYZ**	0,0039	INF

* Fold change was calculated by category (Lame and Control) using Scaffold v.4.8.6 as the ratio between the average of quantitative values in the lame group (numerator) and the control group (denominator). INF indicates zero quantitative values in the control group.

**Fig 1:**
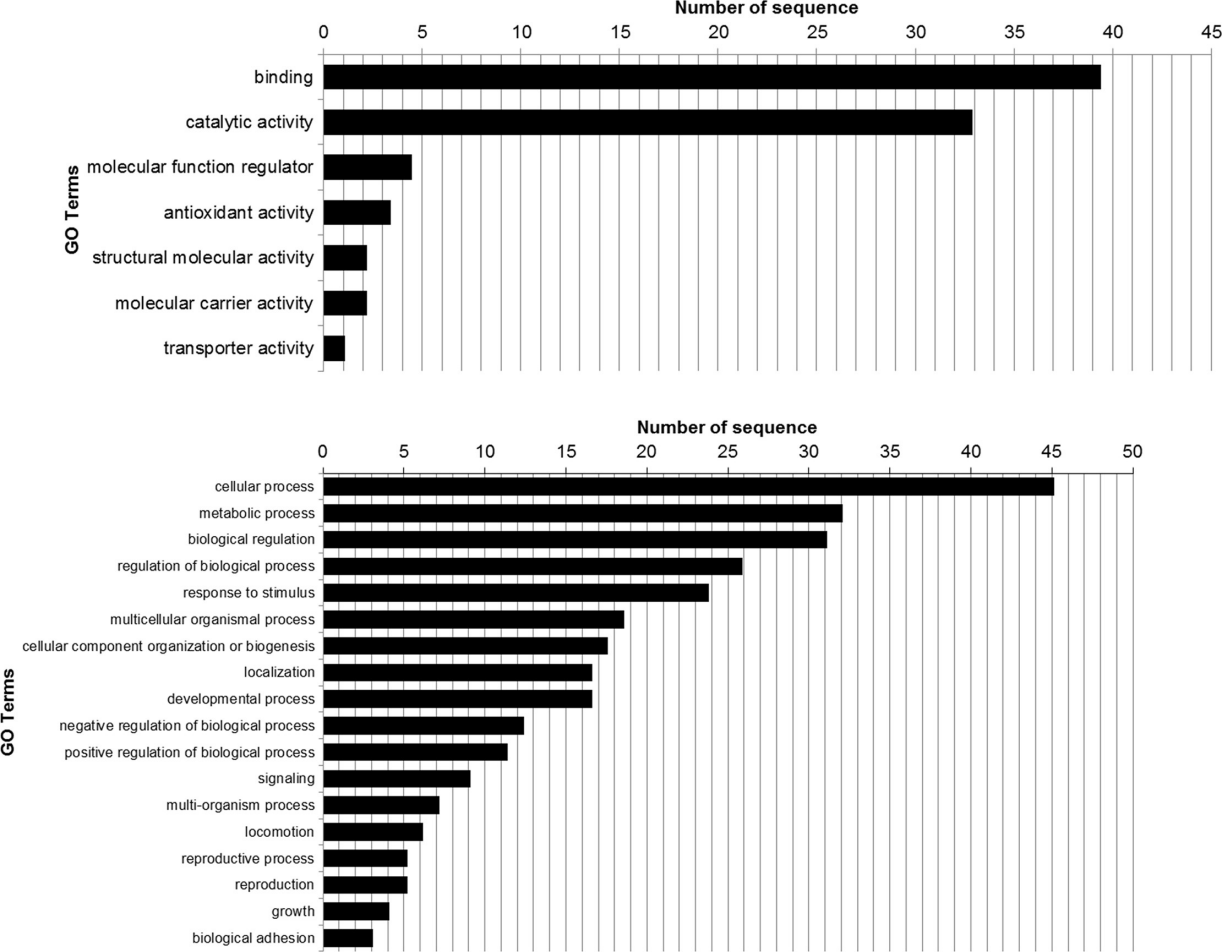
GO annotation for A) molecular function and B) biological process of identified proteins in the dorsal horn of the spinal cord of chronically lame cows.

Gene ontology (GO) annotations were performed in order to obtain an overall biological and functional background of the quantified proteins. Sequence distribution by GO level is shown in Fig 1. GO analysis indicate that proteins were distributed by activity as binding (39.5%), catalytic (32.4%) followed by antioxidant (3.4%) structural (2.2%), carrier (2.2%) and transport (1.1%). Regarding biological process distribution, cellular processes (45.2%), metabolic processes (32.1%) and biological regulation (31.1%) were the most frequent annotated distributions. Gene ontology for cellular localization showed that proteins were primary distributed in cell parts (42,2%) and organelles (31,2%), followed by protein-containing complex (17.9%), membranes (11.6%), membrane enclosed lumen (10.5%), with few sequences distributed at the extracellular region (8.4%), cell junction (2.1%) and nucleoid (1.1%) levels (Fig 1).

**Fig 2:**
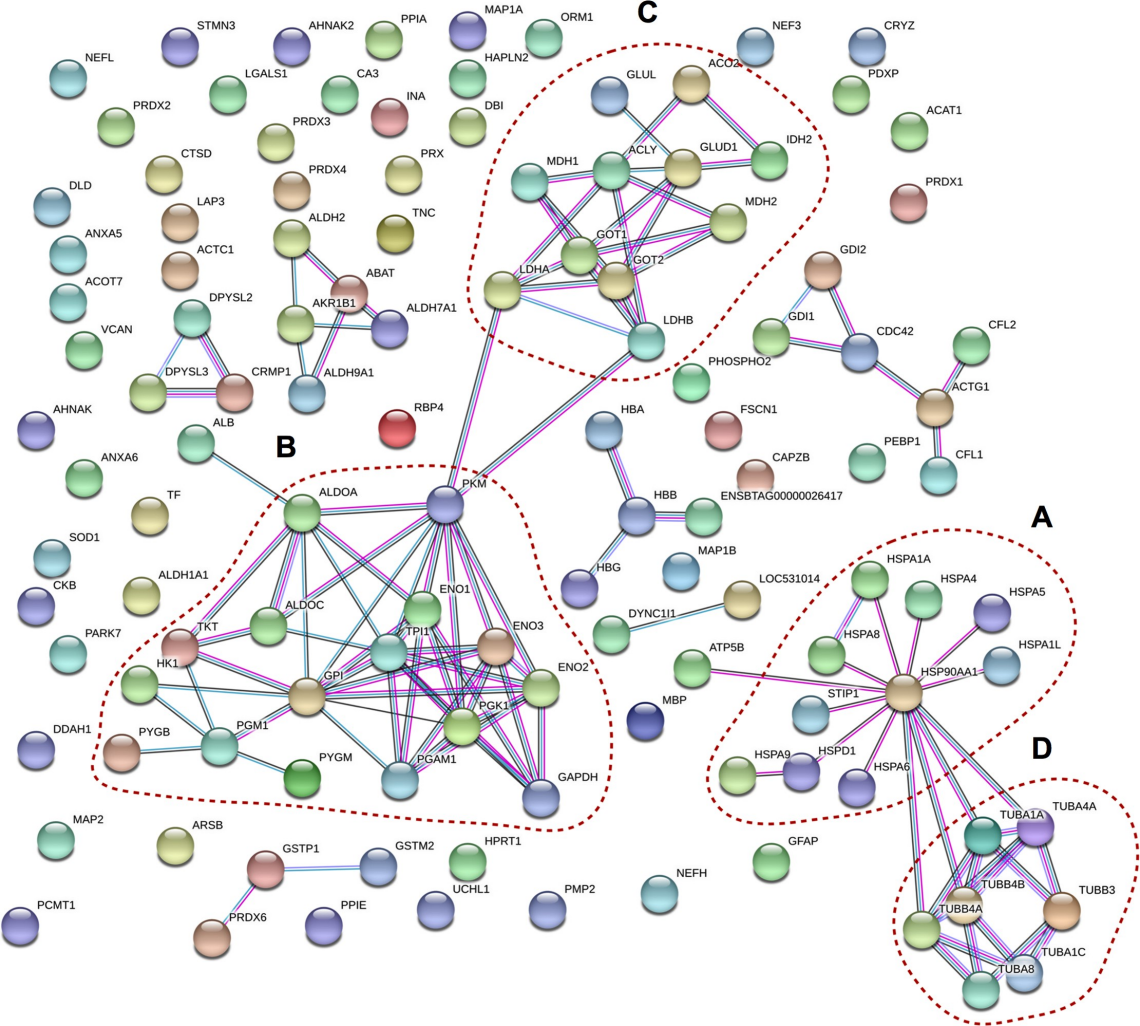
STRING network graphic of proteins detected in the spinal cord. Edges represents evidence of the type of interaction, selecting database, experiments co-expression and co-occurrence as active interaction sources with a high confident of interaction score (0.900). Circles distinguish four groups of interaction. **A**: consisted in 10 proteins identified as chaperones and co-chaperones in which 7 were only detected in lame cows and 1 was strongly upregulated in lame cow. **B**: conformed by 16 proteins involved in glycolysis, gluconeogenesis and pentose phosphate pathway. **C**: composed mainly by mitochondrial proteins involved in the tricyclic acid pathway with a relative even expression in both groups. **D**: interaction generated by tubulin isoforms with no difference in the level of expression among groups but interacting with chaperone network. Light blue edges represent known interactions obtained from curated data base, violet edges represent known interactions experimentally determined, and black edges represent protein association by co-expression.

The identified and quantified proteins were subjected to STRING interaction analysis. Out of 129 proteins, 121 were successfully recognized in the STRING database and represented as a network of proteins connected with evidence-based edges. The resulting network shows four evident clusters in which one protein is connected to at least three other proteins with highest confidence bond (Fig 2). One cluster consisted of 10 proteins identified as chaperones and co-chaperones from which 7 were only detected in lame cows (Fig 2A). A second cluster consisted of 16 proteins associated to glycolysis, gluconeogenesis and the pentose phosphate pathway (Fig 2B), from which 5 of them were upregulated in lame cows (Table 3). Also, a third cluster (Fig 2C) consisting of mitochondrial proteins and a fourth cluster (Fig 2D), consisting of tubulin isoforms showed no differences between lame and non-lame cows. A marked interaction between clusters A and D (Fig 2) could be observed. The Kyoto Encyclopedia of Genes and Genome (KEGG) pathways most representative in the interacting network are depicted in Table 3. The most notorious finding is that antigen processing and presentation pathway (KEGG pathway ID 0416) was only integrated by proteins strongly upregulated in lame cows.

**Table 3:** KEGG pathway ID of interconnected proteins obtained from STRING database.

KEGG Pathway ID	Pathway Description	count in genes	*p* value	Proteins
00010	Glycolysis	18	1.66e-24	ENO1 (↑), ENO2 (↑), ENO3 (↑), GAPD (↓), HK1 (↑), GPI (↔), ALDOA (↔), PGK1 (↔), PGAM1 (↔), LDH (↔)
00020	Citrate cycle (TCA cycle)	6	7.21e-07	ACLY (↑), ACO2 (↑), DLD (↑↑), IDH2 (↔), MDH1 (↔), MDH2 (↔)
04612	Antigen processing and presentation	7	4.15e-06	HSP90AA1 (↑↑), HSP90AB1 (↔), HSPA1 (↑↑), HSPA1L (↑↑), HSPA4 (↑↑), HSPA6 (↑↑), HSPA8 (↑)
00030	Pentose phosphate pathway	5	8.4e-06	ALDOA (↔), ALDOC (↔), GPI (↔), PGM1 (↔), TKT (↑↑)
04066	HIF-1 signalling pathway	5	0.00276	ENO1 (↑), ENO2 (↑), ENO3 (↑), HK1 (↑), TF (↑)

↑Increased expression in lame cows, ↑↑Only detected in lame cows, ↓Reduced expression in lame cows, ↔no difference in expression

**Fig 3:**
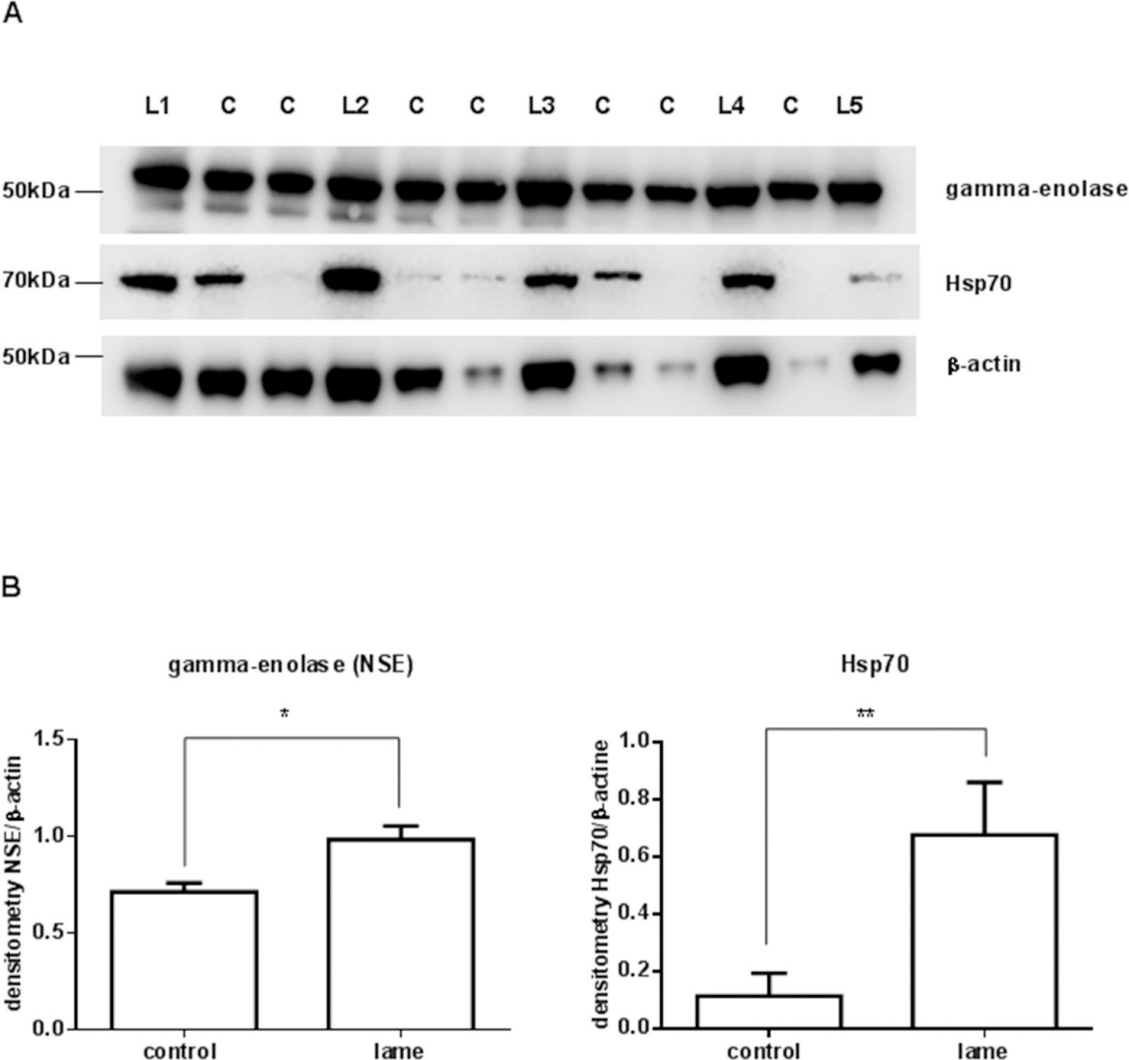
A) Representative western blot of gamma-enolase and Hsp70 from the spinal cord obtained from lame and control cows. B) Densitometric analysis of western blot showing gamma-enolase and Hsp70 upregulation in the spinal cord of lame cows. L: lame (n = 5), C: control (n = 7).

Validation of LC-MS/MS results was performed by western blot analysis of one protein from the chaperone cluster (Hsp70) and one protein from the glycolytic enzymes cluster (gamma-enolase) (Fig 3).

## Discussion

In this study we describe the differential expression of proteins in the dorsal horn of the spinal cord of cows with chronic painful inflammatory lameness using a proteomic analysis. Peptide spectra were identified using SEQUEST search engine and validated through X!Tandem using different modification parameter in order avoid identification redundancy. The totality of the lame dairy cows included in our study had a history of lameness of more than 5 months with notorious and evident hyperalgesia and allodynia of the affected limb during the mobility score evaluation. Lame cows had a higher number of expressed proteins in the dorsal horn of the spinal cord compared to sound cows. Different proteomic profile studies in dorsal root ganglia and spinal cord in various animal models of pain have previously reported an increased number of upregulated proteins compared to downregulated proteins [37].

We describe an increased amount of stress-associated proteins in the spinal cord of lame cows. A 20-time fold change in the constitutive Hsc70 (HSPA8) protein was observed in lame cows, with the inducible forms of Hsp70 (HSPA1L) and Hsp90 (HSPA90AA1) only detected in lame cows. Similar to our findings, several proteomic profiles in chronic neuropathic pain in rodents have reported increasing levels of heat shock proteins (HSP´s) after injury in both the peripheral and central nervous system [37, 38, 39]. Additionally, upregulation of HSP’s has been reported after experimental lower lumbar and sacral terminal nerve compression in dogs [40]. HSP’s are a family of intracellular chaperones that bind to different proteins facilitating their folding into their native and active conformation [41]. In response to cellular stress, upregulation of HSP’s help to protect cells from abnormal protein aggregation, thus preventing cell death [42, 43]. However, newer roles of HSP´s have been described recently, including antigen presentation, immune cell activation and inflammation [44]. Furthermore, neurons, glial and immune cells can secrete stress proteins under specific conditions [45, 46]. In humans, plasma concentration of Hsp90 has been associated with tumor malignancy [47], and similarly, a positive correlation between plasma Hsp70 and plasma cytokines has been described [48]. Also, glial cells can secrete Hsp70 and Hsp90 [49], which can be recognized by several surface receptor including: TLR2/TLR4 [50], CD40 [51] and CCR5 [52]. Interestingly, CD40 and CCR5 are involved in microglial activation and chronic pain development [53, 54]. Moreover, during nerve damage, HSP´s are release from damaged neurons acting as damage-associated molecular patterns (DAMP’s), which can trigger glial and immune cell activation through the TLR2 and TLR4 pathways, contributing to neuropathic pain development [55]. Hsp90 can induce microglial activation in the spinal cord, enhancing hyperalgesia through TLR4 receptor activation [56]. Recently, Nascimento et al. (2018) [57] reported a marked upregulation of Hsp90 in DRG in a rat arthritic model, describing that the pharmacological inhibition of Hsp90 induced analgesia and a reduction in astrocyte activation. Furthermore, increased levels of Hsp90 have been described in different chronic inflammatory diseases [58] and Hsp90 has been associated with the development of neurodegenerative diseases due to its role in stabilizing the transcriptional factor NF-κB [59].

The increased number of chaperone proteins here described could be associated with endoplasmic reticulum (ER) stress inducing an unfolding protein response (UPR) [43, 60]. ER stress promotes cell protection against different insults by altering the transcriptome and proteome. However, prolonged ER stress may disrupt the protective mechanisms of UPR, leading to the activation of inflammatory and apoptotic processes [43]. In our study, the ER chaperone Grp78 (also known as BiP or HSPA5) was strongly upregulated in lame cows. Grp78 has been described as a marker of ER stress and UPR [61]. UPR is associated with an increased demand of protein folding and secretion in the ER, which could explain the elevated number of chaperone proteins found in lame cows. UPR has been reported in several neuroinflammatory conditions in which protein aggregates are found [43]. Recently, Zhou et al. (2017) [62] reported an increased expression of Grp78 in the dorsal horn of the spinal cord in a rat model of inflammatory pain. Increased levels of Grp78 have also been reported in an orofacial pain model in rats [63] and in the spinal cord after L5-spare nerve ligation (SNL) in a rat model of neuropathic pain [64]. Interestingly, increased expression of Grp78 has been shown to participate in the activation of astrocytes and microglia [65, 66]. Moreover, chronic morphine administration causes an upregulation of Grp78 and UPR leading to morphine tolerance in rats [67]. This finding may explain in part the mechanisms involved in the lack of analgesic effect of opioids during chronic pain states or might be involved in the downregulation of the endorphin antinociceptive descending pathway described during chronic pain.

Another cluster of interacting proteins detected in the STRING analysis corresponded to metabolic enzymes. Additionally, GO and KEGG pathway analysis showed a marked number of proteins involved in the glycolytic pathway, tricarboxylic acid pathway and the pentose phosphate pathway. Similar to our findings, changes in the expression of metabolism-associated proteins are frequently reported in pain studies using a proteomic approach [37, 38, 39]. These findings could be explained by the increasing metabolic demand of the neural tissue following nerve damage, since protein synthesis is an energy demanding process [68]. Enolase is a glycolytic and multifunctional enzyme that is frequently detected in proteomic studies [69]. Here, enolase 1 (α-enolase; ENO1), enolase 2 (γ-enolase or neuronal specific enolase; ENO2) and enolase 3 (β-enolase; ENO3) were strongly upregulated in lame cows. Non-glycolytic functions of metabolic enzymes have gained attention, in particular enolase, which can act as plasminogen receptor during pathological states [70]. Inflammatory signals promote enolase translocation to the cell membrane [71] enhancing plasminogen activation [72] and promoting extracellular matrix degradation, metalloproteinase activation, macrophage migration and cytokines synthesis [71, 73]. Cell surface enolase has been found in neurons, activated microglia [74], astrocytes [73] and different immune cells [71]. The role of enolase in chronic pain was confirmed by Polcyn et al. (2017) [75], after selectively blocking its non-glycolytic functions, thus reducing spinal glial activation and cytokine synthesis in a rat model of spinal cord injury.

Antioxidant proteins SOD1, GST, PRDX1 and PRDX4 were significantly upregulated in lame cows. Different modifications in the expression of antioxidant proteins have been reported in pain studies using proteomics analysis [38, 39]. Reactive oxygen species (ROS) increase in the spinal cord during chronic pain [76] participating in the phosphorylation of the NMDA receptor [77], in the activation of TRPA1 and TRPV1 channels [78] and decreasing GABA release by inhibitory interneurons [79]. Furthermore, ROS signaling is an important regulator of ER stress and UPR [80, 81] and acts as a potent stimulus for chaperone proteins upregulation [82], which is consistent with the findings previously described.

Peroxiredoxin 1 (Prdx1) and Peroxiredoxin 4 (Prdx4) are active mediators of the ROS signaling pathway [83]. Prdx1 has previously been reported to increase during chronic pain [39]. Additionally, Prdx1 can be secreted by non-classical pathway, activating the immune response [84]. In contrast, Prdx4 was only detected in the spinal cord of lame cows suggesting a high demand of antioxidant activity in the ER. Prdx4 is a peroxidase with chaperone functions predominantly localized in the ER that assist protein folding by reducing H_2_O_2_, thus preventing oxidative stress in the ER [85]. Increased levels of Prdx have been associated with chronic inflammatory conditions and neurodegeneration [86]. We believe that Prdx proteins may play an important role in the development of chronic pain of lame cows.

Fascin is another protein that was strongly upregulated in the dorsal horn of lame cows. Fascin is an actin-bundling protein that regulates cell motion [87]. Fascin upregulation in the CNS promotes microglial activation; cell migration and proinflammatory cytokine release [88]. Similarly, dihydropyrimidinase-like proteins (Dpysl) participate in synaptic formation during CNS development [89] interacting with actin and microtubules [90]. Two proteins of this family were differentially expressed in lame cows. Dpysl2 was downregulated and Dpysl3 was strongly upregulated. Dpysl2 are frequently downregulated in proteomics studies after nerve damage in order to control axonal guidance [26]. In contrast, microglial activation leads to an increase in the expression of Dpysl3 following stimulation by LPS [91]. Additionally, microtubule-associated protein 1A (MAP1A) and 1B (MAP1B) were only present in lame cows. Both proteins are expressed along the axon and dendritic processes of neurons, both in the CNS and the peripheral nervous system (PNS), where they bind to microtubules and microfilaments [92]. MAP1B is only expressed during embryogenesis and its role in nervous system development has been described [93, 94]. A potential role of MAP1B in the mature CNS has not been defined, but its upregulation could be associated with the presence of protein aggregates in neurodegenerative disease, proteosomal degradation and autophagy [94, 95].

A limitation of our study is that several upregulated proteins in lame cows were not integrated in the enrichment pathway analysis. This could be explained by the incomplete *Bos taurus* annotation database due to the reduced scientific information of the species and to the highest confidence score selected in order to reduce false positive rate. This must be taken into account when bioinformatics analyses are performed in less common species. Also, the small sample size in the lame group must be taken into account before extrapolating these results. Furthermore, the proteomic results here presented must be confirmed using additional functional molecular analysis.

## Conclusions

The results here presented demonstrate that persistent pain originated by chronic inflammatory lameness in dairy cows is partly mediated by ER stress. Evident changes in chaperones, metabolic and redox proteins that are frequently upregulated under cellular stress are described. Moreover, the proteome of the dorsal horn from chronically lame cows showed increased expression of several proteins with non-canonical functions. This non-canonical function might be triggered by stress signals originated from the persistent painful stimulus. Reactive oxygen species, ER stress and UPR are known to play an important role in the maintenance of chronic pain states. Further molecular analysis is necessary in order to confirm the findings here described.

## Supporting information

S1 Data (XLSX)

## References

[1] Latremoliere A, Woolf CJ. Central sensitization: a generator of pain hypersensitivity by central neural plasticity. J Pain. 2009;10: 895–926. 10.1016/j.jpain.2009.06.012 19712899 PMC2750819

[2] Kuner R. Spinal excitatory mechanisms of pathological pain. Pain. 2015;156 Suppl 1: S11–7. 10.1097/j.pain.0000000000000118 25789427

[3] Grandin T. Welfare Problems in Cattle, Pigs, and Sheep that Persist Even Though Scientific Research Clearly Shows How to Prevent Them. Animals (Basel). 2018;8 10.3390/ani8070124 30037055 PMC6071130

[4] Mogil JS, Davis KD, Derbyshire SW. The necessity of animal models in pain research. Pain. 2010;151: 12–17. 10.1016/j.pain.2010.07.015 20696526

[5] Klinck MP, Mogil JS, Moreau M, Lascelles BDX, Flecknell PA, Poitte T, et al Translational pain assessment: could natural animal models be the missing link? Pain. 2017;158: 1633–1646. 10.1097/j.pain.0000000000000978 28614187

[6] Kossaibati MA, Esslemont RJ. The costs of production diseases in dairy herds in England. Vet J. 1997;154: 41–51. Available: https://www.ncbi.nlm.nih.gov/pubmed/9265852 10.1016/s1090-0233(05)80007-3 9265852

[7] Booth CJ, Warnick LD, Gröhn YT, Maizon DO, Guard CL, Janssen D. Effect of lameness on culling in dairy cows. J Dairy Sci. 2004;87: 4115–4122. 10.3168/jds.S0022-0302(04)73554-7 15545373

[8] Garbarino EJ, Hernandez JA, Shearer JK, Risco CA, Thatcher WW. Effect of lameness on ovarian activity in postpartum holstein cows. J Dairy Sci. 2004;87: 4123–4131. 10.3168/jds.S0022-0302(04)73555-9 15545374

[9] Griffiths BE, Grove White D, Oikonomou G. A Cross-Sectional Study Into the Prevalence of Dairy Cattle Lameness and Associated Herd-Level Risk Factors in England and Wales. Front Vet Sci. 2018;5: 65 10.3389/fvets.2018.00065 29675419 PMC5895762

[10] Ranjbar S, Rabiee AR, Gunn A, House JK. Identifying risk factors associated with lameness in pasture-based dairy herds. J Dairy Sci. 2016;99: 7495–7505. 10.3168/jds.2016-11142 27394954

[11] Sepúlveda-Varas P, Lomb J, von Keyserlingk MAG, Held R, Bustamante H, Tadich N. Claw horn lesions in mid-lactation primiparous dairy cows under pasture-based systems: Association with behavioral and metabolic changes around calving. J Dairy Sci. 2018;101: 9439–9450. 10.3168/jds.2018-14674 30100516

[12] Woolf CJ, Ma Q. Nociceptors—noxious stimulus detectors. Neuron. 2007;55: 353–364. 10.1016/j.neuron.2007.07.016 17678850

[13] Chen L, Yang G, Grosser T. Prostanoids and inflammatory pain. Prostaglandins Other Lipid Mediat. 2013;104–105: 58–66. 10.1016/j.prostaglandins.2012.08.006 22981510

[14] Couture R, Harrisson M, Vianna RM, Cloutier F. Kinin receptors in pain and inflammation. Eur J Pharmacol. 2001;429: 161–176. Available: https://www.ncbi.nlm.nih.gov/pubmed/11698039 10.1016/s0014-2999(01)01318-8 11698039

[15] Mizumura K, Murase S. Role of nerve growth factor in pain. Handb Exp Pharmacol. 2015;227: 57–77. 10.1007/978-3-662-46450-2_4 25846614

[16] Zhang N, Inan S, Cowan A, Sun R, Wang JM, Rogers TJ, et al A proinflammatory chemokine, CCL3, sensitizes the heat- and capsaicin-gated ion channel TRPV1. Proc Natl Acad Sci U S A. 2005;102: 4536–4541. 10.1073/pnas.0406030102 15764707 PMC555471

[17] Jin X, Gereau RW 4th. Acute p38-mediated modulation of tetrodotoxin-resistant sodium channels in mouse sensory neurons by tumor necrosis factor-alpha. J Neurosci. 2006;26: 246–255. 10.1523/JNEUROSCI.3858-05.2006 16399694 PMC6674296

[18] Sun WH, Chen CC. Roles of Proton-Sensing Receptors in the Transition from Acute to Chronic Pain. J Dent Res. 2016;95: 135–142. 10.1177/0022034515618382 26597969

[19] Sandkühler J. Models and mechanisms of hyperalgesia and allodynia. Physiol Rev. 2009;89: 707–758. 10.1152/physrev.00025.2008 19342617

[20] Tadich N, Tejeda C, Bastias S, Rosenfeld C, Green LE. Nociceptive threshold, blood constituents and physiological values in 213 cows with locomotion scores ranging from normal to severely lame. Vet J. 2013;197: 401–405. 10.1016/j.tvjl.2013.01.029 23499542

[21] Santos SD, Carvalho AL, Caldeira MV, Duarte CB. Regulation of AMPA receptors and synaptic plasticity. Neuroscience. 2009;158: 105–125. 10.1016/j.neuroscience.2008.02.037 18424006

[22] Chen B-S, Roche KW. Regulation of NMDA receptors by phosphorylation. Neuropharmacology. 2007;53: 362–368. 10.1016/j.neuropharm.2007.05.018 17644144 PMC2001266

[23] Khoutorsky A, Price TJ. Translational Control Mechanisms in Persistent Pain. Trends Neurosci. 2018;41: 100–114. 10.1016/j.tins.2017.11.006 29249459 PMC6004100

[24] Zhang Y, Fonslow BR, Shan B, Baek M-C, Yates JR 3rd. Protein analysis by shotgun/bottom-up proteomics. Chem Rev. 2013;113: 2343–2394. 10.1021/cr3003533 23438204 PMC3751594

[25] Gomez-Varela D, Barry AM, Schmidt M. Proteome-based systems biology in chronic pain. J Proteomics. 2019;190: 1–11. 10.1016/j.jprot.2018.04.004 29653266

[26] Niederberger E, Kühlein H, Geisslinger G. Update on the pathobiology of neuropathic pain. Expert Rev Proteomics. 2008;5: 799–818. 10.1586/14789450.5.6.799 19086860

[27] Mao J. Current challenges in translational pain research. Trends Pharmacol Sci. 2012;33: 568–573. 10.1016/j.tips.2012.08.001 22959652 PMC3482290

[28] Serada S, Fujimoto M, Ogata A, Terabe F, Hirano T, Iijima H, et al iTRAQ-based proteomic identification of leucine-rich alpha-2 glycoprotein as a novel inflammatory biomarker in autoimmune diseases. Ann Rheum Dis. 2010;69: 770–774. 10.1136/ard.2009.118919 19854709

[29] Bäckryd E, Ghafouri B, Carlsson AK, Olausson P, Gerdle B. Multivariate proteomic analysis of the cerebrospinal fluid of patients with peripheral neuropathic pain and healthy controls—a hypothesis-generating pilot study. J Pain Res. 2015;8: 321–333. 10.2147/JPR.S82970 26170714 PMC4492642

[30] Wåhlén K, Olausson P, Carlsson A, Ghafouri N, Gerdle B, Ghafouri B. Systemic alterations in plasma proteins from women with chronic widespread pain compared to healthy controls: a proteomic study. J Pain Res. 2017;10: 797–809. 10.2147/JPR.S128597 28435317 PMC5388344

[31] Tadich N, Flor E, Green L. Associations between hoof lesions and locomotion score in 1098 unsound dairy cows. Vet J. 2010;184: 60–65. 10.1016/j.tvjl.2009.01.005 19211281

[32] Reader JD, Green MJ, Kaler J, Mason SA, Green LE. Effect of mobility score on milk yield and activity in dairy cattle. J Dairy Sci. 2011;94: 5045–5052. 10.3168/jds.2011-4415 21943755

[33] Nesvizhskii AI, Keller A, Kolker E, Aebersold R. A statistical model for identifying proteins by tandem mass spectrometry. Anal Chem. 2003;75: 4646–4658. Available: https://www.ncbi.nlm.nih.gov/pubmed/14632076 10.1021/ac0341261 14632076

[34] Keller A, Purvine S, Nesvizhskii AI, Stolyar S, Goodlett DR, Kolker E. Experimental protein mixture for validating tandem mass spectral analysis. OMICS. 2002;6: 207–212. 10.1089/153623102760092805 12143966

[35] Ashburner M, Ball CA, Blake JA, Botstein D, Butler H, Cherry JM, et al Gene ontology: tool for the unification of biology. The Gene Ontology Consortium. Nat Genet. 2000;25: 25–29. 10.1038/75556 10802651 PMC3037419

[36] Szklarczyk D, Franceschini A, Wyder S, Forslund K, Heller D, Huerta-Cepas J, et al STRING v10: protein-protein interaction networks, integrated over the tree of life. Nucleic Acids Res. 2015;43: D447–52. 10.1093/nar/gku1003 25352553 PMC4383874

[37] Kang SK, So HH, Moon YS, Kim CH. Proteomic analysis of injured spinal cord tissue proteins using 2-DE and MALDI-TOF MS. Proteomics. 2006;6: 2797–2812. 10.1002/pmic.200500621 16586436

[38] Komori N, Takemori N, Kim HK, Singh A, Hwang S-H, Foreman RD, et al Proteomics study of neuropathic and nonneuropathic dorsal root ganglia: altered protein regulation following segmental spinal nerve ligation injury. Physiol Genomics. 2007;29: 215–230. 10.1152/physiolgenomics.00255.2006 17213366

[39] Huang H-L, Cendan C-M, Roza C, Okuse K, Cramer R, Timms JF, et al Proteomic profiling of neuromas reveals alterations in protein composition and local protein synthesis in hyper-excitable nerves. Mol Pain. 2008;4: 33 10.1186/1744-8069-4-33 18700027 PMC2525634

[40] Cízková D, Lukácová N, Marsala M, Kafka J, Lukác I, Jergová S, et al Experimental cauda equina compression induces HSP70 synthesis in dog. Physiol Res. 2005;54: 349–356. Available: https://www.ncbi.nlm.nih.gov/pubmed/15974836 15974836

[41] Yon JM. Protein folding in the post-genomic era. J Cell Mol Med. 2002;6: 307–327. 10.1111/j.1582-4934.2002.tb00511.x 12417049 PMC6740087

[42] van Noort JM. Stress proteins in CNS inflammation. J Pathol. 2008;214: 267–275. 10.1002/path.2273 18161755

[43] Chakrabarti A, Chen AW, Varner JD. A review of the mammalian unfolded protein response. Biotechnol Bioeng. 2011;108: 2777–2793. 10.1002/bit.23282 21809331 PMC3193940

[44] Calderwood SK, Mambula SS, Gray PJ Jr, Theriault JR. Extracellular heat shock proteins in cell signaling. FEBS Lett. 2007;581: 3689–3694. 10.1016/j.febslet.2007.04.044 17499247

[45] Guzhova I, Kislyakova K, Moskaliova O, Fridlanskaya I, Tytell M, Cheetham M, et al In vitro studies show that Hsp70 can be released by glia and that exogenous Hsp70 can enhance neuronal stress tolerance. Brain Res. 2001;914: 66–73. Available: https://www.ncbi.nlm.nih.gov/pubmed/11578598 10.1016/s0006-8993(01)02774-3 11578598

[46] Mambula SS, Stevenson MA, Ogawa K, Calderwood SK. Mechanisms for Hsp70 secretion: crossing membranes without a leader. Methods. 2007;43: 168–175. 10.1016/j.ymeth.2007.06.009 17920512 PMC2745244

[47] Wang X, Song X, Zhuo W, Fu Y, Shi H, Liang Y, et al The regulatory mechanism of Hsp90alpha secretion and its function in tumor malignancy. Proc Natl Acad Sci U S A. 2009;106: 21288–21293. 10.1073/pnas.0908151106 19965370 PMC2795546

[48] Peraçoli JC, Bannwart-Castro CF, Romao M, Weel IC, Ribeiro VR, Borges VTM, et al High levels of heat shock protein 70 are associated with pro-inflammatory cytokines and may differentiate early- from late-onset preeclampsia. J Reprod Immunol. 2013;100: 129–134. 10.1016/j.jri.2013.08.003 24051131

[49] Tytell M, Lasek RJ, Gainer H. Axonal maintenance, glia, exosomes, and heat shock proteins. F1000Res. 2016;5 10.12688/f1000research.7247.1 26962444 PMC4765724

[50] Asea A, Rehli M, Kabingu E, Boch JA, Bare O, Auron PE, et al Novel signal transduction pathway utilized by extracellular HSP70: role of toll-like receptor (TLR) 2 and TLR4. J Biol Chem. 2002;277: 15028–15034. 10.1074/jbc.M200497200 11836257

[51] Wang Y, Kelly CG, Karttunen JT, Whittall T, Lehner PJ, Duncan L, et al CD40 is a cellular receptor mediating mycobacterial heat shock protein 70 stimulation of CC-chemokines. Immunity. 2001;15: 971–983. Available: https://www.ncbi.nlm.nih.gov/pubmed/11754818. 10.1016/s1074-7613(01)00242-4 11754818

[52] Floto RA, MacAry PA, Boname JM, Mien TS, Kampmann B, Hair JR, et al Dendritic cell stimulation by mycobacterial Hsp70 is mediated through CCR5. Science. 2006;314: 454–458. 10.1126/science.1133515 17053144

[53] Cao L, Palmer CD, Malon JT, De Leo JA. Critical role of microglial CD40 in the maintenance of mechanical hypersensitivity in a murine model of neuropathic pain. Eur J Immunol. 2009;39: 3562–3569. 10.1002/eji.200939657 19750482 PMC2810130

[54] Laudati E, Currò D, Navarra P, Lisi L. Blockade of CCR5 receptor prevents M2 microglia phenotype in a microglia-glioma paradigm. Neurochem Int. 2017;108: 100–108. 10.1016/j.neuint.2017.03.002 28279751

[55] Milligan ED, Watkins LR. Pathological and protective roles of glia in chronic pain. Nat Rev Neurosci. 2009;10: 23–36. 10.1038/nrn2533 19096368 PMC2752436

[56] Hutchinson MR, Ramos KM, Loram LC, Wieseler J, Sholar PW, Kearney JJ, et al Evidence for a role of heat shock protein-90 in toll like receptor 4 mediated pain enhancement in rats. Neuroscience. 2009;164: 1821–1832. 10.1016/j.neuroscience.2009.09.046 19788917 PMC2783248

[57] Nascimento DSM, Potes CS, Soares ML, Ferreira AC, Malcangio M, Castro-Lopes JM, et al Drug-Induced HSP90 Inhibition Alleviates Pain in Monoarthritic Rats and Alters the Expression of New Putative Pain Players at the DRG. Mol Neurobiol. 2018;55: 3959–3975. 10.1007/s12035-017-0628-x 28550532

[58] Tukaj S, Węgrzyn G. Anti-Hsp90 therapy in autoimmune and inflammatory diseases: a review of preclinical studies. Cell Stress Chaperones. 2016;21: 213–218. 10.1007/s12192-016-0670-z 26786410 PMC4786535

[59] Bohonowych JE, Hance MW, Nolan KD, Defee M, Parsons CH, Isaacs JS. Extracellular Hsp90 mediates an NF-κB dependent inflammatory stromal program: implications for the prostate tumor microenvironment. Prostate. 2014;74: 395–407. 10.1002/pros.22761 24338924 PMC4306584

[60] Sprenkle NT, Sims SG, Sánchez CL, Meares GP. Endoplasmic reticulum stress and inflammation in the central nervous system. Mol Neurodegener. 2017;12: 42 10.1186/s13024-017-0183-y 28545479 PMC5445486

[61] Wang M, Wey S, Zhang Y, Ye R, Lee AS. Role of the unfolded protein response regulator GRP78/BiP in development, cancer, and neurological disorders. Antioxid Redox Signal. 2009;11: 2307–2316. 10.1089/ARS.2009.2485 19309259 PMC2819800

[62] Zhou F, Zhang W, Zhou J, Li M, Zhong F, Zhang Y, et al Involvement of endoplasmic reticulum stress in formalin-induced pain is attenuated by 4-phenylbutyric acid. J Pain Res. 2017;10: 653–662. 10.2147/JPR.S125805 28360534 PMC5365334

[63] Yang ES, Bae JY, Kim TH, Kim YS, Suk K, Bae YC. Involvement of endoplasmic reticulum stress response in orofacial inflammatory pain. Exp Neurobiol. 2014;23: 372–380. 10.5607/en.2014.23.4.372 25548537 PMC4276808

[64] Zhang E, Yi M-H, Shin N, Baek H, Kim S, Kim E, et al Endoplasmic reticulum stress impairment in the spinal dorsal horn of a neuropathic pain model. Sci Rep. 2015;5: 11555 10.1038/srep11555 26109318 PMC4479804

[65] Kakimura J, Kitamura Y, Taniguchi T, Shimohama S, Gebicke-Haerter PJ. Bip/GRP78-induced production of cytokines and uptake of amyloid-beta(1–42) peptide in microglia. Biochem Biophys Res Commun. 2001;281: 6–10. 10.1006/bbrc.2001.4299 11178952

[66] Mháille AN, McQuaid S, Windebank A, Cunnea P, McMahon J, Samali A, et al Increased expression of endoplasmic reticulum stress-related signaling pathway molecules in multiple sclerosis lesions. J Neuropathol Exp Neurol. 2008;67: 200–211. 10.1097/NEN.0b013e318165b239 18344911

[67] Liu D, Zhou Y, Peng Y, Su P, Li Z, Xu Q, et al Endoplasmic Reticulum Stress in Spinal Cord Contributes to the Development of Morphine Tolerance. Front Mol Neurosci. 2018;11: 72 10.3389/fnmol.2018.00072 29559889 PMC5845556

[68] Lindqvist LM, Tandoc K, Topisirovic I, Furic L. Cross-talk between protein synthesis, energy metabolism and autophagy in cancer. Curr Opin Genet Dev. 2018;48: 104–111. 10.1016/j.gde.2017.11.003 29179096 PMC5869074

[69] Petrak J, Ivanek R, Toman O, Cmejla R, Cmejlova J, Vyoral D, et al Déjà vu in proteomics. A hit parade of repeatedly identified differentially expressed proteins. Proteomics. 2008;8: 1744–1749. 10.1002/pmic.200700919 18442176

[70] Díaz-Ramos A, Roig-Borrellas A, García-Melero A, López-Alemany R. α-Enolase, a multifunctional protein: its role on pathophysiological situations. J Biomed Biotechnol. 2012;2012: 156795 10.1155/2012/156795 23118496 PMC3479624

[71] Bae S, Kim H, Lee N, Won C, Kim H-R, Hwang Y-I, et al α-Enolase expressed on the surfaces of monocytes and macrophages induces robust synovial inflammation in rheumatoid arthritis. J Immunol. 2012;189: 365–372. 10.4049/jimmunol.1102073 22623332

[72] Bock A, Tucker N, Kelher MR, Khan SY, Gonzalez E, Wohlauer M, et al α-Enolase Causes Proinflammatory Activation of Pulmonary Microvascular Endothelial Cells and Primes Neutrophils Through Plasmin Activation of Protease-Activated Receptor 2. Shock. 2015;44: 137–142. 10.1097/SHK.0000000000000394 25944790 PMC4506257

[73] Haque A, Ray SK, Cox A, Banik NL. Neuron specific enolase: a promising therapeutic target in acute spinal cord injury. Metab Brain Dis. 2016;31: 487–495. 10.1007/s11011-016-9801-6 26847611 PMC4864119

[74] Hafner A, Glavan G, Obermajer N, Živin M, Schliebs R, Kos J. Neuroprotective role of γ-enolase in microglia in a mouse model of Alzheimer’s disease is regulated by cathepsin X. Aging Cell. 2013;12: 604–614. 10.1111/acel.12093 23621429

[75] Polcyn R, Capone M, Hossain A, Matzelle D, Banik NL, Haque A. Neuron specific enolase is a potential target for regulating neuronal cell survival and death: implications in neurodegeneration and regeneration. Neuroimmunol Neuroinflamm. 2017;4: 254–257. 10.20517/2347-8659.2017.59 29423430 PMC5800407

[76] Guedes RP, Bosco LD, Teixeira CM, Araújo ASR, Llesuy S, Belló-Klein A, et al Neuropathic pain modifies antioxidant activity in rat spinal cord. Neurochem Res. 2006;31: 603–609. 10.1007/s11064-006-9058-2 16770731

[77] Gao X, Kim HK, Chung JM, Chung K. Reactive oxygen species (ROS) are involved in enhancement of NMDA-receptor phosphorylation in animal models of pain. Pain. 2007;131: 262–271. 10.1016/j.pain.2007.01.011 17317010 PMC2048490

[78] Nishio N, Taniguchi W, Sugimura YK, Takiguchi N, Yamanaka M, Kiyoyuki Y, et al Reactive oxygen species enhance excitatory synaptic transmission in rat spinal dorsal horn neurons by activating TRPA1 and TRPV1 channels. Neuroscience. 2013;247: 201–212. 10.1016/j.neuroscience.2013.05.023 23707800

[79] Yowtak J, Lee KY, Kim HY, Wang J, Kim HK, Chung K, et al Reactive oxygen species contribute to neuropathic pain by reducing spinal GABA release. Pain. 2011;152: 844–852. 10.1016/j.pain.2010.12.034 21296500 PMC3108328

[80] Sharma HS, Gordh T, Wiklund L, Mohanty S, Sjöquist PO. Spinal cord injury induced heat shock protein expression is reduced by an antioxidant compound H-290/51. An experimental study using light and electron microscopy in the rat. J Neural Transm. 2006;113: 521–536. 10.1007/s00702-005-0405-2 16550329

[81] Eletto D, Chevet E, Argon Y, Appenzeller-Herzog C. Redox controls UPR to control redox. J Cell Sci. 2014;127: 3649–3658. 10.1242/jcs.153643 25107370

[82] Niforou K, Cheimonidou C, Trougakos IP. Molecular chaperones and proteostasis regulation during redox imbalance. Redox Biol. 2014;2: 323–332. 10.1016/j.redox.2014.01.017 24563850 PMC3926111

[83] Netto LES, Antunes F. The Roles of Peroxiredoxin and Thioredoxin in Hydrogen Peroxide Sensing and in Signal Transduction. Mol Cells. 2016;39: 65–71. 10.14348/molcells.2016.2349 26813662 PMC4749877

[84] Riddell JR, Wang X-Y, Minderman H, Gollnick SO. Peroxiredoxin 1 stimulates secretion of proinflammatory cytokines by binding to TLR4. J Immunol. 2010;184: 1022–1030. 10.4049/jimmunol.0901945 20018613 PMC2955897

[85] Tavender TJ, Bulleid NJ. Peroxiredoxin IV protects cells from oxidative stress by removing H2O2 produced during disulphide formation. J Cell Sci. 2010;123: 2672–2679. 10.1242/jcs.067843 20627953 PMC2908052

[86] Park MH, Jo M, Kim YR, Lee C-K, Hong JT. Roles of peroxiredoxins in cancer, neurodegenerative diseases and inflammatory diseases. Pharmacol Ther. 2016;163: 1–23. 10.1016/j.pharmthera.2016.03.018 27130805 PMC7112520

[87] Yamashiro S, Yamakita Y, Ono S, Matsumura F. Fascin, an actin-bundling protein, induces membrane protrusions and increases cell motility of epithelial cells. Mol Biol Cell. 1998;9: 993–1006. Available: https://www.ncbi.nlm.nih.gov/pubmed/9571235 10.1091/mbc.9.5.993 9571235 PMC25324

[88] Kim J-K, Lee S-M, Suk K, Lee W-H. A novel pathway responsible for lipopolysaccharide-induced translational regulation of TNF-α and IL-6 expression involves protein kinase C and fascin. J Immunol. 2011;187: 6327–6334. 10.4049/jimmunol.1100612 22102721

[89] Yamashita N, Goshima Y. Collapsin response mediator proteins regulate neuronal development and plasticity by switching their phosphorylation status. Mol Neurobiol. 2012;45: 234–246. 10.1007/s12035-012-8242-4 22351471

[90] Yu-Kemp H-C, Brieher WM. Collapsin Response Mediator Protein-1 Regulates Arp2/3-dependent Actin Assembly. J Biol Chem. 2016;291: 658–664. 10.1074/jbc.C115.689265 26598519 PMC4705386

[91] Manivannan J, Tay SSW, Ling E-A, Dheen ST. Dihydropyrimidinase-like 3 regulates the inflammatory response of activated microglia. Neuroscience. 2013;253: 40–54. 10.1016/j.neuroscience.2013.08.023 23988434

[92] Mohan R, John A. Microtubule-associated proteins as direct crosslinkers of actin filaments and microtubules. IUBMB Life. 2015;67: 395–403. 10.1002/iub.1384 26104829

[93] Tortosa E, Montenegro-Venegas C, Benoist M, Härtel S, González-Billault C, Esteban JA, et al Microtubule-associated protein 1B (MAP1B) is required for dendritic spine development and synaptic maturation. J Biol Chem. 2011;286: 40638–40648. 10.1074/jbc.M111.271320 21984824 PMC3220481

[94] Villarroel-Campos D, Gonzalez-Billault C. The MAP1B case: an old MAP that is new again. Dev Neurobiol. 2014;74: 953–971. 10.1002/dneu.22178 24700609

[95] Sayas CL, Ávila J. Crosstalk between axonal classical microtubule-associated proteins and end binding proteins during axon extension: possible implications in neurodegeneration. J Alzheimers Dis. 2014;40 Suppl 1: S17–22. 10.3233/JAD-132315 24531158

